# A Generic Platform for Cellular Screening Against Ubiquitin Ligases

**DOI:** 10.1038/srep18940

**Published:** 2016-01-08

**Authors:** Timurs Maculins, Nikki Carter, Thierry Dorval, Kevin Hudson, J. Willem M. Nissink, Ronald T. Hay, Husam Alwan

**Affiliations:** 1 Discovery Sciences, AstraZeneca, Alderley Park, Cheshire, SK10 4TG, UK; 2 Discovery Sciences, AstraZeneca, Darwin Building, Cambridge Science Park, Milton Road, Cambridge, CB4 0WG, UK; 3 Oncology iMED, AstraZeneca, Alderley Park, Cheshire, SK10 4TG, UK; 4 Oncology iMED, Darwin Building, Cambridge Science Park, Milton Road, Cambridge, CB4 0WG, UK; 5 Centre for Gene Regulation and Expression, Sir James Black Centre, College of Life Sciences, University of Dundee, Dow Street, Dundee, DD1 5EH, UK

## Abstract

Ubiquitin signalling regulates most aspects of cellular life, thus deregulation of ubiquitylation has been linked with a number of diseases. E3 ubiquitin ligases provide substrate selectivity in ubiquitylation cascades and are therefore considered to be attractive targets for developing therapeutic molecules. In contrast to established drug target classes, such as protein kinases, GPCRs, hormone receptors and ion channels, ubiquitin drug discovery is in its early stages. This is, in part, due to the complexity of the ubiquitylation pathways and the lack of robust quantitative technologies that allow high-throughput screening of inhibitors. Here we report the development of a Ubiquitin Ligase Profiling system, which is a novel and generic cellular technology designed to facilitate identification of selective inhibitors against RING type E3 ubiquitin ligases. Utilization of this system requires a single co-transfection of cells with assay vectors, thereby enabling readout of E3 ubiquitin ligase catalytic activity within the cellular environment. Therefore, our robust high-throughput screening platform offers novel opportunities for the development of inhibitors against this difficult-to-target E3 ligase enzyme class.

Ubiquitylation is a covalent attachment of ubiquitin (Ub) to the lysine residue(s) of a protein substrate that represents an important post-translational regulation of protein activity, localization and half-life. Ubiquitylation is accomplished by an elaborate enzymatic cascade, in which Ub is first activated by two cellular E1 enzymes, then transferred to ~40 E2 enzymes that interact with more than 600 substrate-presenting E3 ubiquitin ligases that confer substrate specificity. E3 ubiquitin ligases are generally divided into two classes based on their mechanism of action: RING (really interesting new gene) and HECT (homologous to the E6-AP carboxyl terminus) domain-containing E3s^1^. Re-iteration of substrate ubiquitylation may subsequently result in the formation of polyubiquitin (polyUb) chains that form distinct topologies known to serve different functions ultimately defining the cellular fate of the ubiquitylated substrate^2^.

As ubiquitin signalling regulates most aspects of cellular life, deregulation of ubiquitylation has been associated with a number of diseases, which present opportunities for pharmacological modulation of this enzymatic cascade and holds a promise for generation of drugs that would restore normal cellular functioning^3^. Well-known examples of drug development efforts in this area represent molecules that interfere with global cellular ubiquitylation through inhibiting E1 or E2 enzymes^3^; however, a more specific modulation of ubiquitin signalling is best achieved at the level of E3 ubiquitin ligases, as these enzymes transfer Ub from E2 to a specific substrate protein^4^.

A general challenge towards a successful drug development campaign is the generation of a robust and sensitive high-throughput screening (HTS) assay for identification of lead compounds from a diverse chemical library. Drug development against Ub signalling is further complicated by the absence of a defined catalytic domain in the structure of E3 ubiquitin ligases, as Ub transfer from E2 to substrates is achieved through protein-protein interactions. Furthermore, a complex enzymatic ubiquitylation cascade, involving at least three enzymes for substrate ubiquitylation to occur, significantly complicates identification of selective E3 ubiquitin ligase inhibitors and therefore requires a robust screening cascade for identification of lead compounds with a selectivity against E3 enzymes.

Developing robust cell-free assays requires extensive protein purification, laborious characterization of co-factors, substrates and choice of a detection technology for the screening assay. Nevertheless, several HTS assays have been developed for this class of enzymes^5^,^6^. Despite these efforts, drug development against E3 ubiquitin ligases is minimal as compared to some well-explored enzyme families, such as protein kinases^7^. To address these limitations, we developed a Ubiquitin Ligase Profiling (ULP) system, which presents a cellular HTS screening platform for identification of compounds against the RING class of E3 ubiquitin ligases. Using this platform, we demonstrate the application of this technology through the identification of inhibitor compounds against Rnf8 E3 ubiquitin ligase. Validation of the ULP method by biochemical *in vitro* and functional cell-based experiments further highlights the importance of this novel technology as a toolkit in drug discovery against this difficult-to-target class of enzymes.

## Results

**Figure 1:**
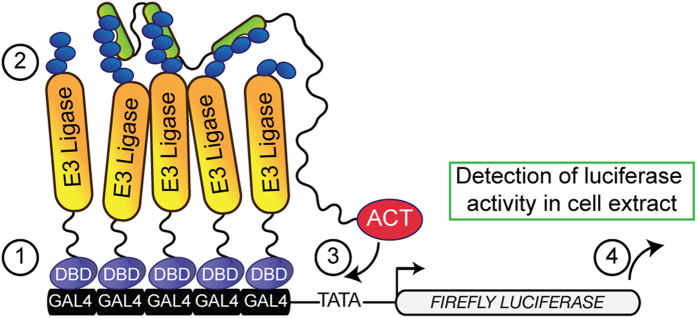
Design of the ULP method. CheckMateTM/Flexi® mammalian two-hybrid vectors modified as indicated in Materials and Methods to express a fusion of the Gal4 DNA-binding domain (DBD, purple) to an E3 ubiquitin ligase (orange), and a fusion of the VP16 activation domain (ACT, red) to TUBEs (green) are co-transfected into mammalian cells along with the firefly luciferase reporter vector, which contains five Gal4 binding sites upstream of the minimal TATA box. Binding of DBD to the Gal4 cassette at the luciferase promoter results in the recruitment of the E3 ubiquitin ligase to the reporter vector (step 1). The E3 ligase autoubiquitylation activity results in decoration of poly-ubiquitin chains (blue ovals) on the ligase surface, creating binding sites for TUBEs (step 2). This subsequently leads to the recruitment of ACT to the minimal TATA box (step 3), thereby linking the E3 catalytic activity under study to the expression of the firefly luciferase reporter, the activity of which is detected following cell lysis (step 4).

**Figure 2:**
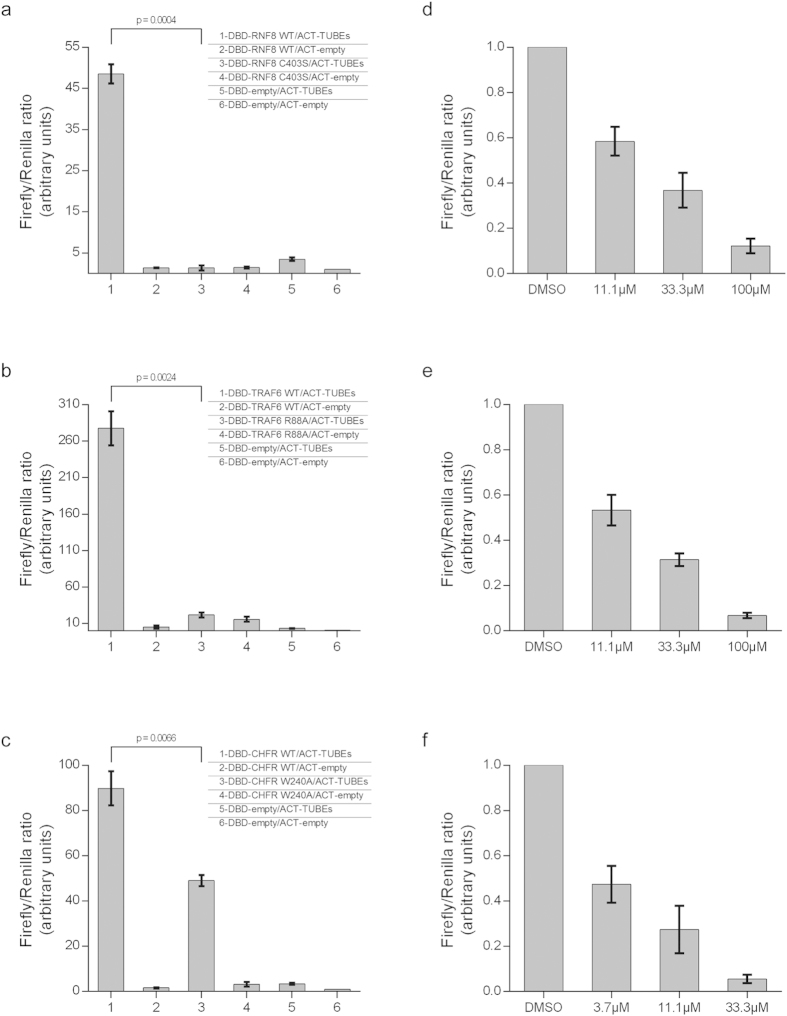
Proof-of-principle of the ULP method. (**a–c**) HEK293 cells co-transfected with (**a**) Rnf8, (**b**) Traf6 or (**c**) Chfr ULP assay vectors as indicated (n = 3). Firefly/Renilla ratio serves as normalization control for transfection efficiency. The background level of luciferase is measured in the presence of empty vector controls and used for data normalization. E3 ligase mutants are used to demonstrate assay dependence on the catalytic activity of E3s. (**d–f**) U-2 OS cells co-transfected with (**a**) Rnf8, (**b**) Traf6 and (**c**) Chfr ULP assay vectors were treated with varying concentrations of Ubc13 inhibitor NSC697923 for two hours before cell lysis to determine the ULP assay activation. Data normalized to DMSO treated controls (n = 3).

To address the current limitations for the development of E3 ubiquitin ligase inhibitors, we designed a novel method for detecting the autocatalytic activity of E3 ubiquitin ligases in cells^8^, thus enabling pharmacological screening. The ULP method is based on a two-hybrid technology^9^, coupling transcriptional reporter activation to ligase autocatalytic activity that is detected by ubiquitin binding domain-based sensors (Fig. 1). These sensors are known as Tandem Ubiquitin Affinity Entities (TUBEs)^10^,^11^ and are widely used for detecting ubiquitylation events^12^,^13^. We first validated the ULP method by detecting autoubiquitylation activity of Rnf8, Traf6 and Chfr ubiquitin ligases in this assay. In contrast to wild type E3 ubiquitin ligases that activated assay signal, co-transfection of cells with catalytically-dead Rnf8 C403S and Traf6 R88A mutants^14^,^15^ abolished assay activation to the background level (Fig. 2a,b). A Chfr W240A mutant was previously reported to have a reduced catalytic activity^16^. Consistently, utilizing this mutant was associated with a significant reduction of assay signal (Fig. 2c). Together this demonstrated that E3 catalytic activity is essential for ULP assay activation. As Rnf8, Traf6 and Chfr ubiquitin ligases predominantly function in concert with the upstream Ubc13 ubiquitin-activating enzyme^14^,^17^,^18^, we subsequently assessed ULP assay response to NSC697923, a covalent inhibitor of the Ubc13 enzyme^19^. As predicted, the ULP assays displayed a concentration-dependent response to Ubc13 inhibition (Fig. 2d–f). Taken together, the above experiments demonstrate that the ULP method enables specific detection of E3 catalytic activity in the cellular context.

**Figure 3:**
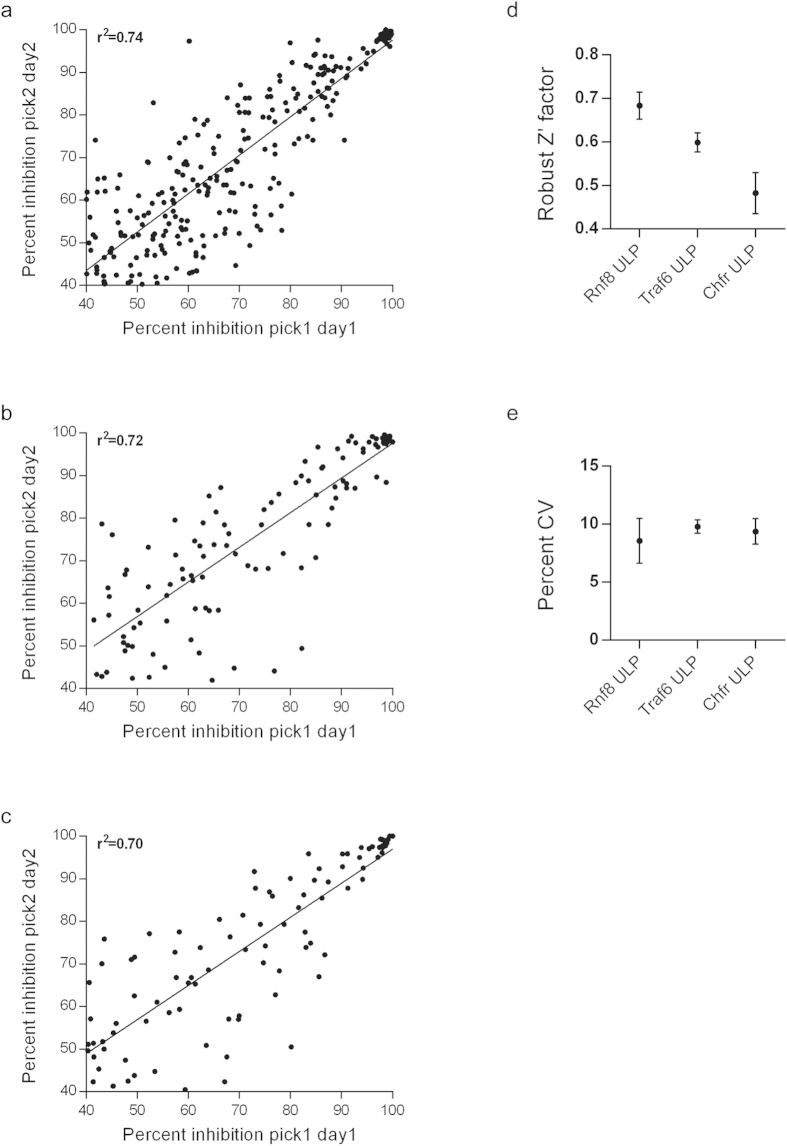
Validation of the ULP method. (**a–c**) (**a**) Rnf8, (**b**) Traf6 and (**c**) Chfr assay-ready cell bank validated with AstraZeneca test set comprised of 9,846 compounds to determine data reproducibility. Cryopreserved assay-ready cells were dispensed into compound-ready 384-well plates on two separate days with different compound well locations (pick1 or pick2). Firefly luciferase activity was determined following 20-hour incubation. Data shown for compounds with ≥40% activity on both occasions. (**d**) Z’ factors of ULP assays (Rnf8 ULP −0.68 ± 0.02 SEM; Traf6 ULP −0.60 ± 0.01 SEM; Chfr ULP −0.48 ± 0.03 SEM). (**e**) Coefficient of variation (CV) of ULP assays (Rnf8 ULP −8.6% ± 1.1 SEM; Traf6 ULP −9.8% ± 0.3 SEM; Chfr ULP −9.4 %± 0.6 SEM).

We then prepared a single batch of cryopreserved assay-ready cells co-transfected with either Rnf8, Traf6 or Chfr ULP assay vector combinations and miniaturized the assay to enable screening. Analysis of the data quality control demonstrated that the ULP assays comply with industry standards for cell-based assays^20^ and thus represent a potential platform for targeting of ubiquitin ligase activity (Fig. 3). To examine the robustness of the ULP method as a pharmacological screening platform we selected Rnf8 ubiquitin ligase as a target for HTS, as it plays a central role in DNA damage response. Rnf8 is recruited rapidly to DNA double strand breaks through phosphorylated Mdc1, a process regulated by the Atm kinase^21^. Rnf8 catalytic activity results in recruitment of DNA double strand break repair factors, such as the 53bp1 protein^22^,^23^, an event that is crucial for DNA repair and genomic integrity in normal and cancer cells. Thus, Rnf8 may constitute a novel target for pharmacological inhibition with potential applications in oncology. We therefore utilized the ULP method in our screening cascade to facilitate hit identification against Rnf8.

**Figure 4:**
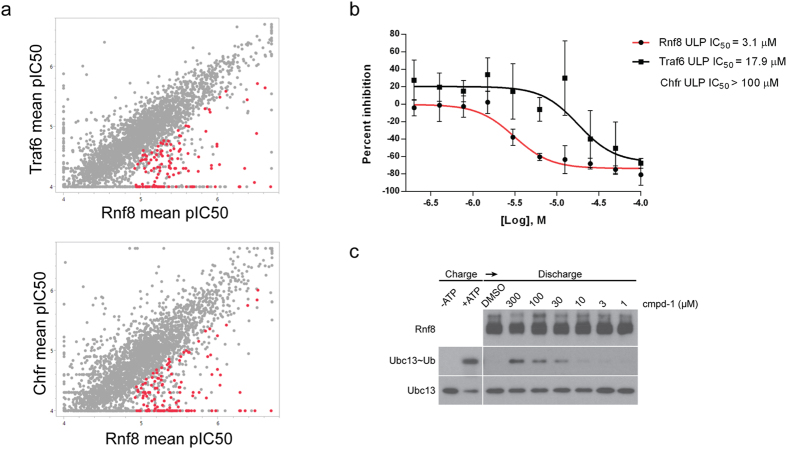
ULP method reveals a subset of Rnf8 selective compounds. (**a**) Mean pIC_50_ values of 3,327 hits compared between Rnf8 and Traf6 assays (upper panel) and between Rnf8 and Chfr assays (lower panel) (n = 3). A subset of 127 hits with Rnf8 ULP pIC_50_ ≥ 4.9 and ≥0.5 log unit selectivity in Rnf8 versus both Traf6 and Chfr assays is highlighted as red dots in both plots. (**b**) Concentration-dependent response of compound-1 in Rnf8, Traf6 and Chfr ULP assays using a concentration range from 0.2–100 μM in two-fold dilution steps (n = 5). Note that compound-1 does not demonstrate Chfr assay inhibition within the tested concentration range (data not shown), and therefore Chfr ULP IC_50_ is indicated as >100 μM. (**c**) Concentration-dependent block of Ubc13~Ub thioester discharge by compound-1 (cmpd-1).

Primary screening using a single compound concentration was conducted with a diverse library comprised of 92,908 compounds, utilizing the previously validated Rnf8 ULP assay-ready cell bank. We identified 3,327 hits that inhibited Rnf8 ULP assay signal by ≥40%. To eliminate off-target compounds that act through inhibiting luciferase expression, affecting cell viability or global ubiquitylation upstream of Rnf8, the identified hits were used for a secondary screening stage in which Rnf8, Traf6 and Chfr ULP assay-ready cells were incubated with 10 concentrations of each compound. This allowed us to determine IC_50_ and compound selectivity. We first used a pre-calculated promiscuity index to assign a confidence score to each of the hits reflecting compound chemical properties and activity based on historic screening data and eliminated frequent hitters^24^ (data not shown). We then determined selectivity margins of hits by calculating the pIC_50_ value differences in Rnf8 versus both Traf6 and Chfr assays. Compounds that displayed a selectivity margin <0.5 log units in Rnf8 versus Traf6 and/or Chfr were deprioritized. Example responses of these compounds are shown in Supplementary Fig.1a,b (excluded compounds marked as grey dots in Fig. 4a). In contrast, compounds that showed ≥0.5 log unit selectivity in Rnf8 versus both Traf6 and Chfr were classified as selective within the context of the current data (Supplementary Fig.1c and Fig. 4a). This analysis identified a subset of 127 Rnf8 hits (marked as red dots in Fig. 4a), demonstrating that application of the ULP method in pharmacological screening with a diverse chemical library enables identification of compounds with selectivity against the targeted E3 ubiquitin ligase of interest.

**Figure 5:**
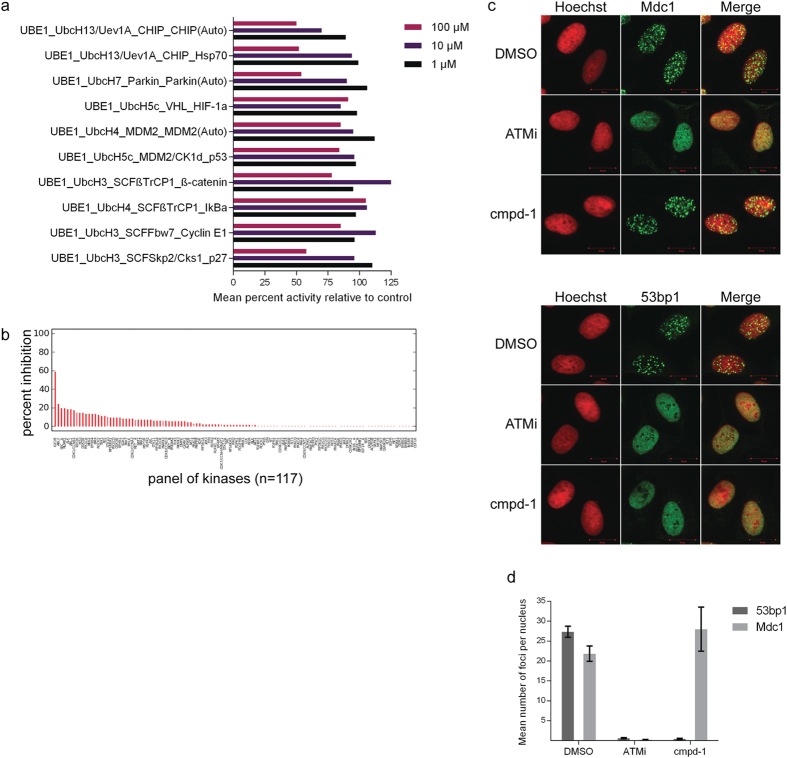
Inhibition of Rnf8-driven signalling by compound-1. (**a**) Mean percent inhibition data (n = 2) for compound-1 across a panel of 10 indicated ubiquitylation cascades at 1, 10 and 100 μM concentration. (**b**) Percent inhibition data for compound-1 across a panel of 117 kinases. Single-shot data was generated for a concentration of 1 μM of compound. Negative values have been truncated to zero in the plots. (**c**) Confocal microscopy of U-2 OS cells pre-incubated with either DMSO, Atm inhibitor (12.5 μM) or compound-1 (50 μM) prior to irradiation with 10 Gy. Repair foci visualized by immunofluorescence with Mdc1 (upper panel) or 53bp1 (lower panel) antibodies and Hoechst for nuclear staining. (**d**) Quantification of data displayed in (**c**).

We then progressed the identified Rnf8 selective subset of hits to biochemical validation by using lysine discharge *in vitro* assay. This assay utilizes a purified ubiquitylation cascade, where Rnf8 serves as the only source of ubiquitin ligase activity^18^. In this ubiquitylation cascade, Ubc13 E2 ubiquitin-conjugating enzyme is initially charged with Ub, resulting in the formation of Ubc13~Ub thioester, which is discharged by addition of Rnf8 E3 ligase and Ube2V2 (a co-factor of Ubc13). Focusing on compound-1, which was highly selective in the ULP assays (Fig. 4b), we utilized the lysine discharge *in vitro* assay to further validate this compound. Indeed, biochemical analysis revealed that compound-1 blocks Ubc13~Ub thioester discharge in a concentration-dependent manner (Fig. 4c). We next examined the effect of compound-1 on other ubiquitylation cascades *in vitro*. We assessed the activity of a panel of 10 different *in vitro* ubiquitylation cascades that utilize a variety of E2 and E3 enzymes in a substrate-dependent or independent manner. We also selected compound-2 that displayed a selectivity margin <0.5 log units against Rnf8 in ULP assays and therefore was anticipated to harbour promiscuous behaviour (Supplementary Fig. S2a). In contrast to the non-specific inhibition mediated by compound-2 (Supplementary Fig. S2b), all tested ubiquitylation cascades showed more than 50% activity even at the highest concentration of compound-1, indicating that compound-1 lacks promiscuous inhibition of other E2 or E3 enzymes (Fig. 5a).

Given the crosstalk between cellular phosphorylation and ubiquitylation^25^, we also examined whether compound-1 exhibits non-specific inhibition of kinases and tested its effect on a panel comprised of 117 human protein kinases. These experiments showed that compound-1 lacks non-specific promiscuous inhibition of kinase activity since all of the kinases, with the exception of the epidermal growth factor receptor 1 kinase (59% inhibition) were active in the presence of compound-1 (Fig. 5b).

Given the non-promiscuous selective nature of compound-1 against Rnf8, we then determined its effect on Rnf8-driven signalling in cells, through assessing the recruitment of Mdc1 (upstream of Rnf8) and 53bp1 (downstream of Rnf8) to DNA damage-induced repair foci by confocal imaging relative to DMSO-treated cells. Since compound-1 showed a preferential inhibition of Rnf8-driven ubiquitylation *in vitro*, we anticipated inhibition of 53bp1, but not Mdc1 foci formation following DNA damage. As a positive control, we used Atm kinase inhibitor that blocks recruitment of both, Mdc1 and 53bp1, to repair foci. DMSO-treated cells displayed no impaired recruitment of both Mdc1 and 53bp1 to DNA damage repair foci. Pre-treatment of cells with an Atm kinase inhibitor before cell irradiation, on the other hand, blocked the requirement of Mdc1 and 53bp1 as reported previously^26^,^27^. Importantly, treatment of cells with compound-1 had no effect on the recruitment of Mdc1 to repair foci, suggesting that it does not harbour off-target inhibition of the Atm pathway even at 50 μM concentration (see upper panel in Fig. 5c). In contrast, compound-1 inhibited the recruitment of 53bp1 to repair foci at the same concentration, demonstrating a specific inhibition of Rnf8-driven signalling in cells (see lower panel in Fig. 5c and quantification in Fig. 5d).

Altogether, the above validation experiments demonstrate that the ULP method is an effective tool for identifying compounds against E3 ubiquitin ligases of interest, thus presenting a novel HTS platform that promises to facilitate the discovery of selective inhibitors against this difficult-to-target class of enzymes.

## Discussion

The lack of robust cellular screening approaches constitutes a major technical challenge and represents a barrier for drug development against E3 ubiquitin ligases. Here, we have utilized ubiquitin binding domain-based sensors for detecting the E3 autocatalytic activity, which enabled us to develop a robust HTS-compatible cellular ULP technology. By applying the ULP method to Rnf8 we identified a subset of compounds that show selectivity towards Rnf8 when compared to Traf6 and Chfr. Focusing on compound-1, we demonstrate that it selectively inhibits the activity of Rnf8, but not other ubiquitylation cascades *in vitro*. Cell-based assays also highlight the ability of compound-1 in selectively inhibiting Rnf8-driven cellular signalling during DNA damage response in cells. Altogether data presented in this manuscript demonstrates the effectiveness of the ULP method as a robust cellular screening platform against E3 ubiquitin ligases.

Several cell-free approaches for detecting the activity of E3 ubiquitin ligases are developed and applied in drug discovery. During the ubiquitylation reaction E3 ubiquitin ligases are positioned in close proximity to substrate proteins, which enables the application of the fluorescence resonance energy transfer technology^28^,^29^. Other biochemical *in vitro* approaches that utilize reconstituted ubiquitylation cascades have also reported identification of E3 inhibitors^30^,^31^, but this approach is only possible for well-characterized E3s and requires laborious deconvolution of hits. Structure-driven approaches also offer powerful tools in identifying potent inhibitors of ubiquitin ligases^4^,^32^. However, this approach is complicated by the absence of defined catalytic domains in the structure of ubiquitin ligases, and thus necessitates high-resolution structures of protein-protein interactions between E3 ubiquitin ligases and their cognate substrates or partner E2 enzymes. Despite the progress achieved by cell-free approaches, pharmacological inhibition of E3 ubiquitin ligases still remains a largely underdeveloped area.

The ULP technology offers a number of advantages over the existing cell-free methods. First, detailed characterization of the ubiquitylation cascades that involve the E3 of interest is not a pre-requisite for designing an ULP-based screening assay. For example, in this study we utilized Chfr ligase that had not yet been extensively characterized at the time of writing this manuscript, yet the ULP generic technology enabled us to generate a robust screening assay for this enzyme. Second, the ULP system enables the rapid identification of selective hits against the targeted E3 ubiquitin ligase since multiple ligases could be screened in parallel using an identical two-hybrid technology, avoiding the need for substrate specific reagents, which may not be readily available or may introduce variations in hit identification. For example, Rnf8 hits identified in the primary screening (~3.6% of tested compounds) were progressed to concentration-response secondary screening using Traf6 and Chfr ULP assay-ready cells, which resulted in rapid deconvolution and identification of a subset of hits with a selectivity margin against Rnf8 (~0.13% of tested compounds). This enables large-scale screening with diverse chemical libraries to rapidly identify a subset of interesting compounds for validation experiments.

Through our manuscript we also demonstrate that autoubiquitylation can be effectively utilized as a readout of the E3 ubiquitin ligase activity in a cellular screening assay. Although the role of Rnf8 autoubiquitylation in cells is yet to be fully elucidated, by utilizing it as a readout we identified compound-1 that effectively blocks Rnf8-driven signalling in response to DNA damage. It will be interesting to determine in the future the mechanism(s) of action and whether compound-1 inhibits ubiquitylation of Rnf8 substrates or affects Rnf8 interaction with partner E2 enzymes.

Data presented in this manuscript, therefore demonstrates that targeting E3 ubiquitin ligases in a cellular setting is possible. The ULP method provides the first generic cell-based assay platform for screening against E3 ubiquitin ligases. This cellular high-throughput screening system offers the unmatched benefits of utilizing different cell lines and disease models to identify compounds that are cell permeable and selectively inhibitory against E3 ubiquitin ligases of interest. We therefore anticipate that the ULP technology will enable drug discovery against multiple therapeutically relevant members of this challenging and yet unexplored enzyme class.

## Methods

### Plasmids

Details of PCR primers and constructs that were used in this study are listed in Supplementary Table S1 and S2 respectively. CheckMate^TM^/Flexi® vector mammalian two-hybrid system (Promega, C9360) was used for the design of the ULP system. The pFN11A (BIND) Flexi® vector was utilized to insert RNF8 cDNA sequence (DU19751, DSTT) using PCR primers 9 and 10; TRAF6 cDNA sequence (DU6915, DSTT) using PCR primers 121 and 122; CHFR cDNA sequence (RC228465, OriGene) using PCR primers 62 and 63. The pFN10A (ACT) Flexi® vector was used to insert TUBEs ORF^11^ (using PCR primers 25 and 26) according to manufacturer’s instructions. RNF8 C403S and TRAF6 R88A point mutations were introduced using site-directed mutagenesis kit (Agilent Technologies, QuikChange II) according to manufacturer’s instructions using combinations of PCR primers 37 and 38 (RNF8 C403S); 172 and 173 (TRAF6 R88A); 182 and 183 (CHFR W240A).

### Low-throughput ULP assays

Low-throughput ULP assays were performed by co-transfection of derivatives of pFN10A, pFN11A described above and pGL4.31 reporter vectors into HEK293 or U-2-OS cell lines in a 6-well plate format using FuGENE® HD transfection reagent (Promega, E2311) according to manufacturer’s instructions. The Dual-Luciferase® Reporter assay system (Promega, E1910) was utilized to determine the activities of firefly (*Photinus pyralis*) and *Renilla* (*Renilla reniformis*) luciferases that were measured sequentially from a single sample according to manufacturer’s instructions using an Envision® plate reader (Perkin Elmer).

### Mammalian cell culture and preparation of assay-ready cell bank

All cell lines were grown under standard culture conditions at 37 °C with 95% humidity in 5% CO_2_ tissue culture incubator. HEK293 cells were cultured and screened in media consisting of Dulbecco’s Modified Eagle’s Medium (DMEM) (Sigma-Aldrich, D5546) containing 10% fetal bovine serum (FBS) (PAA), 1% Glutamax (Invitrogen, 35050–038), 1% non-essential amino acids (Invitrogen, 11140–035) and 10 μg/ml penicillin-streptomycin mix (Sigma-Aldrich, 4333). U-2 OS cells were cultured in Roswell Park Memorial Institute medium (RPMI-1640) (Sigma-Aldrich, R7509), supplemented with 10% FBS, 1% Glutamax, 1% non-essential amino acids and 10 μg/ml penicillin-streptomycin mix.

To prepare a cell bank for HTS HEK293 cells were transiently transfected using MaxCyte STX electroporation device, according to the manufacturer’s instructions with a combination of vectors comprising pTM4, pTM40 or pTM48 together with pTM10 and pGL4.31 firefly luciferase reporter vector. Following electroporation, cells were incubated for 20 minutes in a tissue culture incubator and cryopreserved at 3 × 10^7^/ml in 1 ml aliquots.

### Compound Preparation

All test compounds, including anisomycin minimum control (Sigma-Aldrich, A9789), were solubilized in 100% (v/v) DMSO to 10 mM. To generate assay-ready plates, compounds were acoustically dispensed using an Echo® 555 into white 384-well tissue culture–treated microplates (Greiner Bio-One, 781080) for the luminescence-based reporter assay. For single-concentration screening, 10 nl of standard compound or 50 nl of low molecular weight compound stock was dispensed to prepare assay-ready plates, which were then thermally sealed and stored at room temperature. All single-concentration screening was performed at a final concentration of 0.5% (v/v) DMSO maximum control, 5 μM anisomycin minimum control, a standard compound concentration of 10 μM and low molecular weight compound concentration of 50 μM.

For concentration-response screening, varying volumes of compound stocks were dispensed to generate 10-point, half-log concentration-response curves, with a final highest concentration of 100 μM. Wells were backfilled with the required volume of DMSO to ensure a final concentration of 1% (v/v). Plates containing on-board controls (n = 12) for maximum (0% inhibition of assay response) and minimum (100% inhibition of assay response) compounds were used to determine assay quality and compound activity. For concentration-response experiments, the maximum assay signal was defined with 1% (v/v) DMSO and 5 μM anisomycin as a minimum control.

### High-throughput cellular ULP screen

Cryopreserved cell bank vials were rapidly defrosted in a water bath at 37 °C. For each set, cells were transferred to a 50 ml centrifuge tube containing 10 ml of warm assay media. Cells were then centrifuged at 1200 rpm for 5 minutes, resuspended in pre-warmed assay media at 1 × 10^6^ cells/ml and 10 μl/well were dispensed into assay-ready pates using Thermo Scientific™ Multidrop™ Combi Reagent Dispenser liquid dispenser in the tissue culture class II cabinet. Plates were then lidded and incubated for 20 hours at 37 °C, 5% CO_2_ in a humidified tissue culture incubator in plate stackers. After 20-hour incubation, plates were removed from the incubator and allowed to cool at room temperature for 30 minutes. SteadyGlo® reagent (Promega, E2550) prepared as described according to the manufacturer’s instructions was then added to all wells using a Thermo Scientific™ Multidrop™ Combi Reagent Dispenser (10 μl/well) and incubated at room temperature for 30 minutes in the dark before measuring luminescence on an Envision® plate reader (Perkin Elmer) according to manufacturer’s instructions.

All screening data were analysed using Genedata Screener®, which is a commercial software package developed by Genedata AG, Basel, Switzerland. For primary data we employed a standard 2-point normalization of the data to allow the selection of active compounds using controls on the plate to set the 0 and −100% points. In Genedata Screener® a convention is used whereby inhibitions are denoted by negative % effect values and stimulations by positive % effect values. For the analysis of concentration response data, a 2-point normalization was applied to the data as for the primary screen and the resulting concentration response curves plotted and fitted using the Genedata Smart Fit curve fitting algorithm. The Smart Fit algorithm uses a 4 parameter logistic non-linear curve fit with automated strategies to determine whether points that are statistical outliers on the curve should be removed from the analysis and whether the curve asymptotes should be set at fixed values resulting in either 4, 3 or 2 parameter Hill fits. This algorithm aims to achieve the best curve fit to the data with minimal, potentially subjective, user input. Concentration-dependent response curves presented in the manuscript were fit using robust fit algorithm.

### UBC13~Ub lysine discharge assay

Reactions were performed in the reaction buffer containing 50 mM Tris pH 7.5, 150 mM NaCl, 0.1% NP40, 0.5 mM TCEP. Ube1 (DU32888, 90% purity), Ubc13 (DU15705, 90% purity), Ube2V2 (DU12415, 90% purity) and Rnf8 (DU19751, 80% purity) were acquired from Division of Signal Transduction Therapy, University of Dundee. 2 μM Ubc13 (final concentration (f.c.) in 5 μl of charging reaction) was mixed with Ubiquitin (117 μM f.c., Sigma-Aldrich, U6553) 150 nM E1. ATP (3 mM f.c., Sigma-Aldrich, A2383) and MgCl_2_ (5 mM f.c., Bio Basic Canada Inc, MRB0328) were added to start a charging reaction followed by incubation for 12 minutes at 37 °C in the reaction buffer described above. The charging reaction was terminated by the addition of Apyrase (New England Biolabs, MO393L), followed by a 10 minutes incubation at room temperature. Discharge reactions containing Rnf8 (300 nM f.c.) and/or Ube2V2 (300 nM f.c.) and access L-lysine monohydrochloride (150 mM f.c., Sigma-Aldrich, L8662) were set up on ice in a ubiquitin reaction buffer without ATP. Reactions were initiated by the addition of an equal volume of charged Ubc13~Ub thioester and the discharge mix (10 μl total volume), rapidly mixed, and incubated at room temperature. Aliquots were removed at the indicated times and added to an equal volume of non-reducing Novex® Tris-Glycine SDS Sample Buffer (2X) (Life Technologies, LC2676). Ubc13~Ub thioester, Ubc13, and Rnf8 were analysed by non-reducing SDS-PAGE and immunoblotting with anti-Ubc13 (Abcam, ab25885) and anti-Rnf8 antibodies (SantaCruz, sc-271462). To examine a potential effect of compounds on inhibiting Ubc13~Ub discharge, the discharge mix consisting of 300 nM Rnf8 and 300 nM Ube2V2 was first pre-incubated with test compounds at the indicated final concentrations or DMSO at room temperature for 45 minutes before mixing with charged Ubc13~Ub thioester and processing as described above.

### Immunofluorescence microscopy

U-2 OS cells were grown in 96-well flat bottom tissue culture treated microplates (Falcon®, 353219), treated with compounds at the indicated concentrations for 3 hours before irradiation with 10Gy (X-RAD 320) according to manufacturer’s instructions. Cells were then incubated for 3 hours before fixed in 3.7% formaldehyde for 20 minutes, incubated for 5 minutes with cell permeabilisation buffer (0.25% Triton-X (v/v) in PBS) at room temperature. For fluorescence staining, cells were next blocked in 10% FBS for 40 minutes prior to incubation with either anti-Mdc1 (Abcam, ab11171) or anti-53bp1 (Santa Cruz, sc-22760) primary antibodies (in 1% FBS, 0.05% Triton-X (v/v)) for 1 hour at room temperature. Alexa Fluor® 488 conjugate secondary antibody (Life Technologies, A-11008) was then added for an additional hour at room temperature in the dark before staining Hoechst 33342 (Life Technologies, R37605). Images were acquired and captured using the LSM-710 fluorescent microscope.

### Kinase profiling assays

Protein kinase assays were conducted using the KinaseProfiler^TM^ service of Eurofins Pharma Discovery Services UK Limited and as described previously^33^. The kinase of interest was incubated with the test compound in assay buffer containing substrate, 10 mM magnesium acetate and [γ-^33^P-ATP]. The reaction was initiated by the addition of the Mg/ATP mix. After incubation for 40 minutes at room temperature, the reaction was stopped by the addition of a 3% phosphoric acid solution. An aliquot of the reaction was then spotted onto a filtermat and washed in phosphoric acid followed by a rinse in methanol prior to drying and scintillation counting. Results were expressed in relation to controls containing DMSO only in place of test compound. The ATP concentration in each assay was within 15 μM of the determined apparent *K*_*m*_ for ATP.

Lipid kinase assays were conducted using the KinaseProfiler^TM^ service of Eurofins Pharma Discovery Services UK Limited. The kinase of interest was incubated in assay buffer containing substrate and Mg/ATP. The reaction was initiated by the addition of the Mg/ATP solution. After incubation for 30 minutes at room temperature, the reaction was stopped by the addition of Stop solution containing EDTA and a biotinylated form of the reaction product. Finally, Detection buffer was added, containing europium-labelled anti-GST monoclonal antibody, a GST-tagged lipid binding domain and streptavidin-conjugated allophycocyanin. The plate was then read in time-resolved fluorescence mode and the homogeneous time-resolved fluorescence (HTRF) signal was determined according to the formula HTRF = 10000× (Em665 nm/Em620 nm). Compound-1 profiling was conducted against a panel of 117 human protein kinases at a single concentration of 1 μM. More information about assay protocols used for the kinase profiling are available at http://www.eurofins.com/pharma-services/pharma-discovery-services/services/in-vitro-pharmacology/kinases/biochemical.aspx.

### E3 ubiquitin ligase profiling

Ubiquitin E3 ligase assays were conducted using the UbiquitinProfiler service of Eurofins Pharma Discovery Services UK Limited. The E3 ligase of interest was incubated with the test compound in assay buffer containing ATP, UBE1, a suitable E2 conjugating enzyme, Myc-tagged substrate and biotinylated-ubiquitin. The reaction was initiated with the addition of biotinylated-ubiquitin. After incubation for 30 minutes at room temperature, the reaction was terminated by the addition of EDTA-containing Stop solution. Reaction products were separated by capture onto a microplate coated with anti-c-Myc antibody and washing with PBS containing 0.05% Tween 20. E3 ligase activity was measured by detection of bound ubiquitin via electrochemiluminescence. Results were expressed in relation to controls containing DMSO only in place of test compound. Compound-1 and compound-2 profiling against 10 ubiquitylation cascades was conducted at three concentrations of 1, 10 and 100 μM against cascades 1, 2, 5, 6, 9, 10, 16, 17, 20, 21. The assay protocols used for the kinase profiling are available at http://www.eurofins.com/pharma-services/pharma-discovery-services/services/in-vitro-pharmacology/ubiquitin/e3-ligases.aspx.

## Additional Information

**How to cite this article**: Maculins, T. *et al.* A Generic Platform for Cellular Screening Against Ubiquitin Ligases. *Sci. Rep.* **6**, 18940; doi: 10.1038/srep18940 (2016).

## Supplementary Material

Supplementary Information
