# Supplementary material for: A Generic Platform for Cellular Screening Against Ubiquitin Ligases
Source: Sci Rep. 2016 Jan 8;6:18940. doi: 10.1038/srep18940 (PMC4705515; doi:10.1038/srep18940)
Supplement: Supplementary Information [file srep18940-s1.pdf]

A Generic Platform for Cellular Screening Against Ubiquitin Ligases

Authors

Timurs Maculins, Nikki Carter, Thierry Dorval, Kevin Hudson, J. Willem M. Nissink, Ronald T. Hay and Husam Alwan.

Supplementary Figure S1

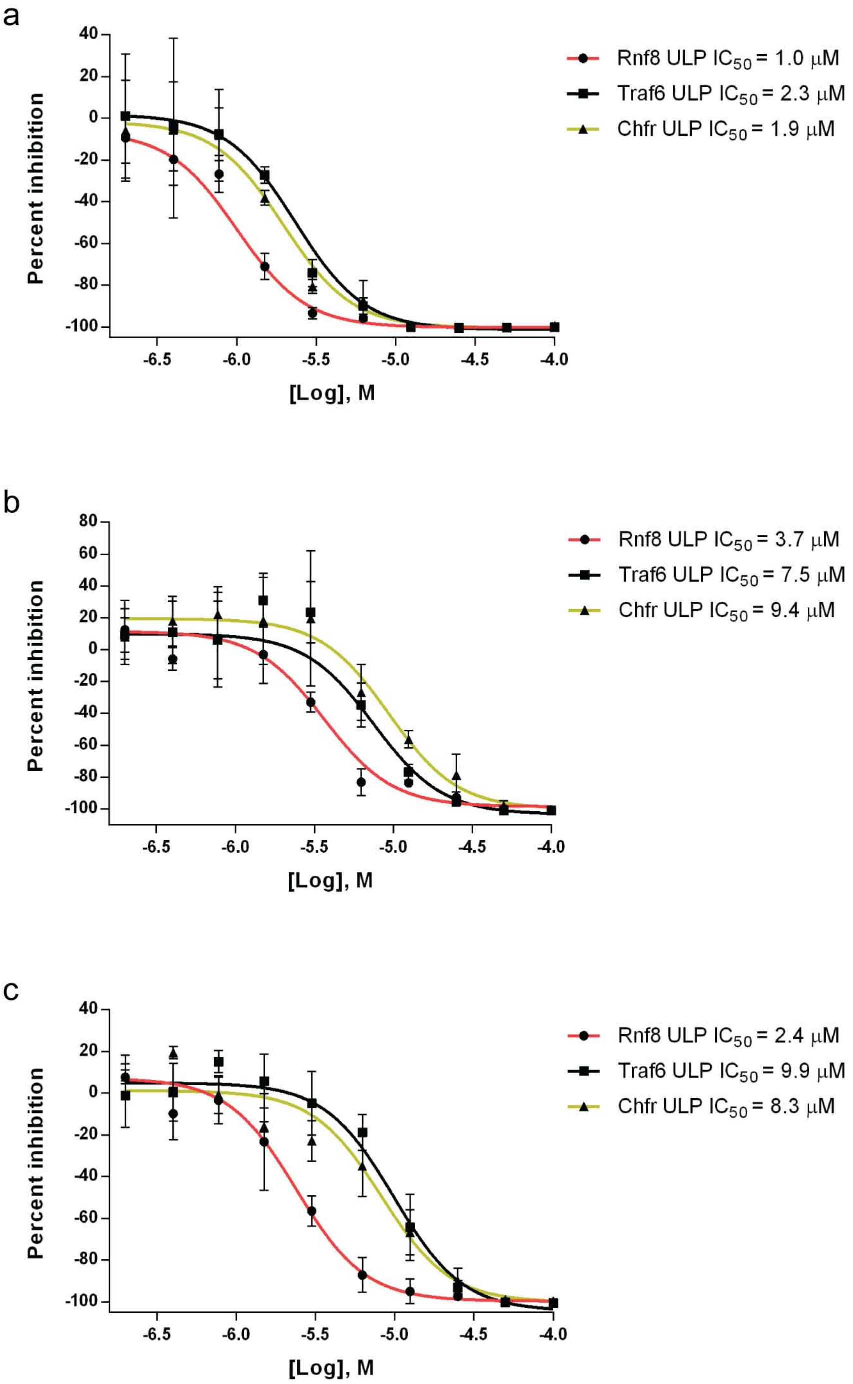

Supplementary Figure S1. Examples of compounds with different selectivity profiles. Dose-response curves for (**a**) a deprioritized compound lacking preferential inhibition of the Rnf8 ULP assay, (**b**) a compound exhibiting selectivity for Rnf8 ULP assay less than 0.5 log units and (**c**) a compound exceeding our minimal selectivity threshold of 0.5 log units against Rnf8 ULP assay (n=5).

Supplementary Figure S2

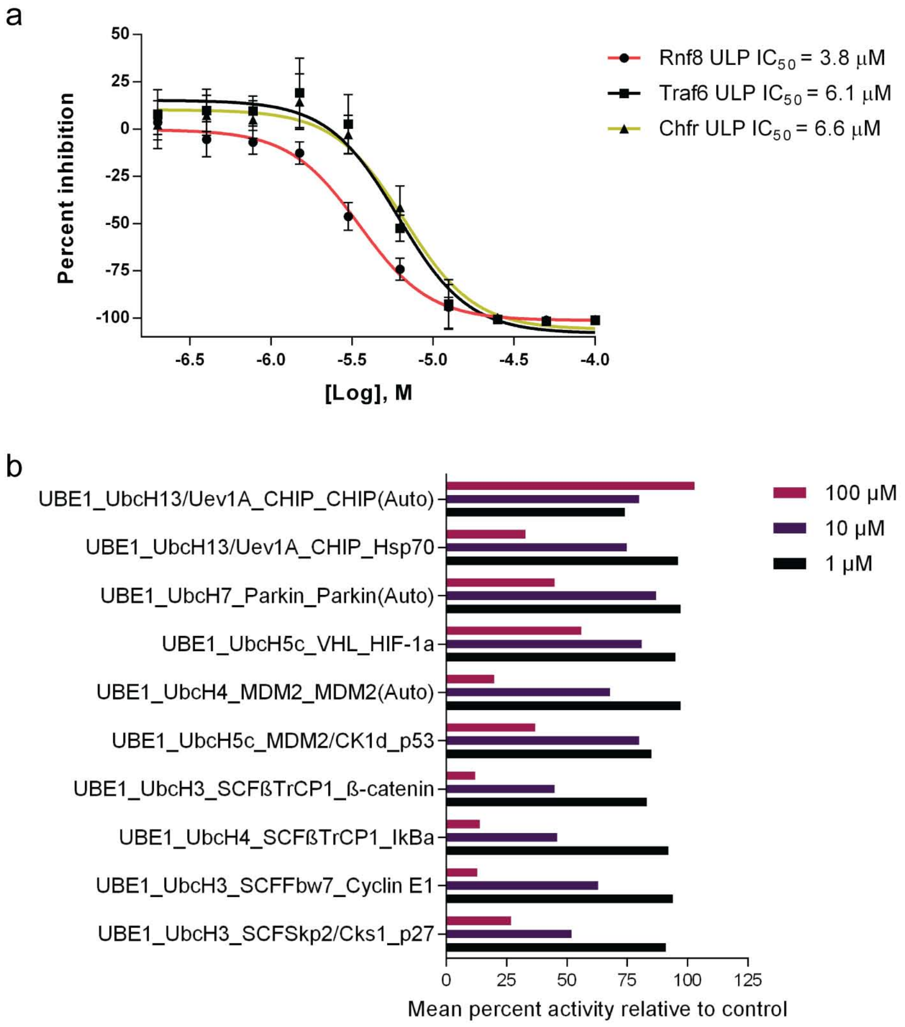

Supplementary Figure S2. Non-specific effect of compound-2. (**a**) Compound lacking preferential inhibition of Rnf8 in the ULP assays (n=5). (**b**) Compound-2 exhibits non-selective inhibition of the majority of indicated ubiquitylation cascades *in vitro* (n=2).

Supplementary Table S1. Details of PCR primers used in this study.

Primer	Description	Primer sequence
9	SgfI RNF8 5'	GACCGCGATCGCCATGGGGGAGCCCGGCTTCTT
10	PmeI RNF8 3'	GTCGGTTTAAACTCAGAACAATCTCTTTGCTTTTCGTTCCC
25	SgfI 3XUIM-7A 5’	CACCGCGATCGCCATGACAGAGGAGGAACAGTTCGCCCTTG
26	PmeI 3XUIM-7A 3’	CTTGGTTTAAACCTAGGAAGGCCTACAGGAATTCAGGGATTCTGC
37	RNF8 t1207a FW	TGTGCTAGAGAATGAGCTCCAAAGTATTATTTGTTCAGAATACTTC
38	RNF8 t1207a RV	GAAGTATTCTGAACAAATAATACTTTGGAGCTCATTCTCTAGCACA
62	SgfI-CHFR 5'	CACGGCGATCGCCATGGAGCGGCCCGAGGAAGGC
63	PmeI-CHFR 3'	TTCTGTTTAAACTTAGTTTTTGAACCTTGTCTGTTCACAGAT
121	SgfI-TRAF6 5'	TTGTGCGATCGCCATGAGTCTGCTAAACTGTGAA
122	PmeI-TRAF6 3'	GGGGGTTTAAACTCATACCCCTGCATCAGTACTT
172	TRAF6 R88A FW	ACGCCATGCGGCCATGCGTTCTGCAAAGCCTG
173	TRAF6 R88A RV	CAGGCTTTGCAGAACGCATGGCCGCATGGCGT
182	FW CHFR W240A	CTTGCTACTCGGGCGCGATGGAGCGCTCGT
183	RV CHFR W240A	ACGAGCGCTCCATCGCGCCCGAGTAGCAAG

Supplementary Table S2. Details of constructs used in this study.

Construct name	Vector Backbone	Insert Description
pTM4	pFN11A	SgfI-PmeI RNF8
pTM10	pFN10A	SgfI-PmeI TUBEs
pTM20	pFN11A	SgfI-PmeI RNF8 C403S
pTM40	pFN11A	SgfI-PmeI CHFR
pTM48	pFN11A	SgfI-PmeI TRAF6
pTM61	pFN11A	SgfI-PmeI CHFR W240A
pTM63	pFN11A	SgfI-PmeI TRAF6 R88A
pBIND	pFN11A	Empty control vector

pACT	pFN10A	Empty control vector
Reporter Vector	pGL4.31	Firefly Luciferase
